# Variation p.R1045H in *MYH7* correlated with hypertrophic cardiomyopathy in a Chinese pedigree

**DOI:** 10.1186/s12920-021-01046-2

**Published:** 2021-07-30

**Authors:** Yan Zhang, Yiyi Shang, Luo Liu, Xiaoxue Ding, Haiyan Wu, Lijiang Li, Mingjie Pang

**Affiliations:** 1 Department of Magnetic Resonance Imaging, The First People’s Hospital of Yunnan Province, The Affiliated Hospital of Kunming University of Science and Technology, Kunming, Yunnan Province China; 2 Department of Cardiology, The First People’s Hospital of Yunnan Province, The Affiliated Hospital of Kunming University of Science and Technology, No. 157 Jinbi Road, Kunming, 650032 Yunnan Province China

**Keywords:** Pedigree analysis, Hypertrophic cardiomyopathy, *MYH7*, Whole-exome sequencing

## Abstract

**Background:**

Inherited hypertrophic cardiomyopathy (HCM) is a common heart muscle disease that damages heart function and may cause the heart to suddenly stop beating. Genetic factors play an important role in HCM. Pedigree analysis is a good way to identify the genetic defects that cause disease.

**Methods:**

An HCM pedigree was determined in Yunnan, China. Whole-exome sequencing was performed to identify the genetic variants of HCM. Another 30 HCM patients and 200 healthy controls were also used to investigate the frequency of the variants by customized TaqMan genotyping assay.

**Results:**

The variant NM_000257.4:c.3134G > A (NP_000248.2:p.Arg1045His, rs397516178, c.3134G > A in short) was found to cosegregate with the clinical phenotype of HCM. Moreover, the variant was not found in the 200 control subjects. After genotyping the variant in 30 HCM patients, there was one patient who carried the variant and had a family history.

**Conclusions:**

Our findings suggest that this variant may be closely related to the occurrence of the disease. According the ACMG guidelines, the c.3134G > A variant should be classified as "Likely pathogenic".

## Background

Inherited cardiomyopathy (ICM) is a heritable disease with abnormal cardiac structure and function. Numerous disease-causing genes for different cardiomyopathies have been found during the past two decades [1]. Inherited hypertrophic cardiomyopathy (HCM) is a form of ICM with thickened heart muscles and is caused by genetic mutations [2]. The main pathological manifestation of inherited HCM is asymmetric left ventricular hypertrophy, which is also a frequent cause of sudden death in this disease [3]. The latest epidemiological investigation shows that the incidence of HCM cases is approximately 1:500 in developed countries. Only 50–60% of these cases can be linked to hereditary cases, among those where no mutation is found hereditary cases also exist, and there is no difference between sexes [4].

Some patients do not show obvious clinical manifestations, which results in difficulty in identifying the disease initially and delays the best treatment opportunities for the patients. At present, there are medical and surgical treatments available that reduce symptoms and prevent sudden cardiac death, so early diagnosis of the disease becomes very important. Genetic diagnosis is an effective application for the early prediction of disease occurrence [5]. Thus, both the U.S [6]. and Europe [7] have established genetic evaluations of cardiomyopathy, so it is valuable to study the mutations of pathogenic genes in HCM.

A previous report confirmed that many pathogenic variants of hypertrophic cardiomyopathy are found in genes encoding sarcomere proteins [2], of which the gene encoding cardiac β-myosin heavy chain (*MYH7*) is a hotspot gene with pathogenic mutations [8]. MYH7 is the predominant motor protein in cardiomyocytes. The *MYH7* gene, located on chromosome 14q12, is mainly expressed in ventricular muscle, atrial muscle and slow skeletal muscle, such as the musculus soleus, in humans. This gene was the first pathogenic gene related to the pathogenesis of HCM to be discovered. Approximately 30–50% of mutation-positive patients (who account for only approximately half of all HCM patients) show mutations in *MYH7* [9].

Although there has been much research on the molecular genetics of HCM in recent years, research on the correlation between *MYH7* gene variants and HCM is still insufficient in China [10, 11]. In this study, we found a pedigree with an "uncertain significance" variant of *MYH7*. All the patients carried the variant, and the other family members did not. To determine the relationship between this variant and the disease, we conducted comprehensive analyses of the family and investigated the incidence of this variant in Chinese HCM patients and controls. Finally, characteristics of the variant were evaluated using the American College of Medical Genetics (ACMG) criteria [12].

## Methods

### Sample information

**Fig. 1:**
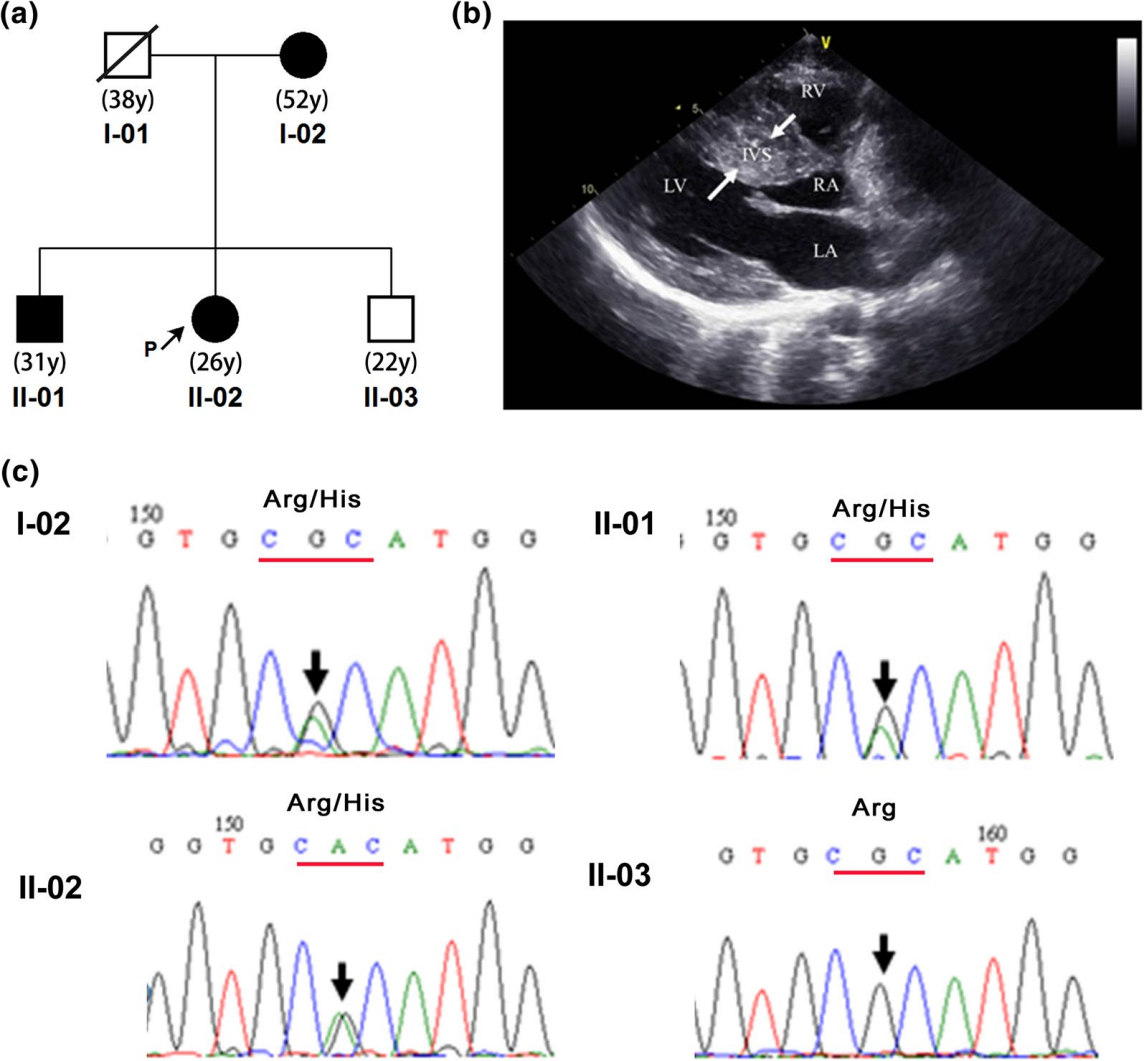
The information of the HCM pedigree. **a** The pedigree map of the HCM family in this study. II-02 is the proband. **b** Apical four-chamber view of patient II-02, showing a markedly thickened ventricular septum. RV: right ventricle, LV: left ventricle, IVS: ventricular septum, RA: right atrium, LA: left atrium. **c** The results of Sanger sequencing to verify the variant found by whole-exome sequencing. Partial electropherograms of the genomic region covering the *MYH7* gene, with the representation showing the coding strand. Patients I-02, II-01, and II-02, which carry variant NM_000257.3: c.1370 T > G (p.Ile457Arg) in a heterozygous state. II-03 is an unaffected relative

A pedigree with HCM was determined (Fig. 1a). The proband (II-02), her older brother (II-01) and their mother (I-02) were diagnosed with HCM. In this family, HCM displays autosomal dominant inheritance. The peripheral blood of four persons in this pedigree was collected. Furthermore, 30 patients with HCM were enrolled. According to the inquiry survey, only two of these 30 patients had a family history of heart disease.

The clinical diagnosis of HCM referred to the 2020 American Heart Association standards [13]: echocardiography indicates that the left ventricular wall thickness or interventricular septum thickness is more than or equal to 15 mm; other causes of myocardial hypertrophy, such as hypertension, rheumatic heart disease, mitral valve disease, congenital heart disease (atrial septum, ventricular septal defect) and myocardial hypertrophy accompanied by metabolic diseases, were excluded. Additionally, 200 healthy people were recruited for the control group. These people did not have heart disease or other diseases. Their blood pressure was normal, and they had no family history of heart disease. All research subjects were of Chinese Han descent. All the patients and controls provided written informed consent, and the study was approved by the Ethics Committee of The First People’s Hospital of Yunnan Province.

### Blood samples and DNA extraction

Three milliliters of peripheral blood was collected from the four pedigree members, HCM patients and 200 controls into EDTA anticoagulation tubes. The samples were stored at −80 °C for later use. A genomic DNA extraction kit (No. 9765, TaKaRa, Japan) was used to extract DNA from 250 μl peripheral blood.

### Next-generation sequencing of HCM pedigree members

Whole-exome sequencing was performed on the 4 members of the HCM pedigree (Fig. 1a). Their genomic DNA was fragmented to an average size of 180–280 bp and used for library creation with the Agilent SureSelect Human All Exon V6 Kit (Agilent Technologies, Santa Clara, CA, USA). The Illumina NovaSeq 6000 platform (Illumina Inc., San Diego, CA, USA) was used for genomic DNA sequencing at Novogene Bioinformatics Technology Co., Ltd. (Beijing, China). Next, 150 bp paired-end reads were generated. The 100 × coverage and 60 million reads were as expected.

After sequencing, bcl2fastq software (Illumina) was used for base calling. After removing low-quality reads, the fastq files were aligned to the reference human genome (hs37d5) using Burrows-Wheeler Aligner (BWA) [14]. Single-nucleotide variants (SNVs) and indels were identified with SAMtools to generate gVCF files [15].

### Sanger sequencing

The p.Arg1045His variant found in the HCM pedigree was sequenced by Sanger sequencing in the four pedigree members to confirm the finding in the exome. The variant was located at position 23,891,500 on chromosome 14 (chr14: 23,891,500, GRCh37.p13).

The PCR primers were designed by Primer3 [16]. They were MYH7F: GCTGTCTTGGGTCTG CTTGT and MYH7R: GGTTTCCCAAGTCCTGAACA. The designed PCR product was 328 bp long. PCR was performed using the following conditions: 94 °C for 3 min; 94 °C for 30 s; annealing at 54 °C for 30 s; and extension at 72 °C for 30 s for a total of 40 cycles. PCR products were purified by a DNA gel recovery kit (TsingKe, China). The purified PCR products were sequenced on a 3130 Sequencer (Applied Biosystems, USA) by using the BigDye Terminator v1.1 Cycle Sequencing Kit (Applied Biosystems, USA) [17]. Following the manufacturer’s instructions, 4 μL BigDye Terminator v1.1 Ready Reaction Mix, 3.2 pmol MYH7F (or MYH7R) and 10 ng purified PCR products were mixed and added to water to reach a total volume of 20 µl. The mixture was subjected to cycle sequencing, purified and loaded into the capillary of a 3130 Sequencer.

### TaqMan custom SNP genotyping assays

TaqMan custom SNP genotyping assays were used to analyze the genotype of the variant found in the HCM pedigree in 200 controls and 30 HCM patients. TaqMan Genotyping Master Mix, SNP Genotyping Assay and genomic DNA were used in combination following the protocol of the TaqMan Custom SNP Genotyping Assay Kit (Applied Biosystems, USA). Real-time PCR was performed on the QuantStudio™ 3 Real-Time PCR System (Applied Biosystems, USA). The data were analyzed by TaqMan Genotyper Software (Applied Biosystems, USA).

## Results

### Clinical phenotype of patients

In the HCM pedigree of this study, the proband (II-02, 26-years-old) had dyspnea, shortness of breath and occasional syncope. Cardiac ultrasound showed that the apical four-chamber view of the proband had a markedly thickened ventricular septum (Fig. 1b). The other two patients (I-02, 52-years-old and II-01, 31-years-old) in the pedigree also had thickened ventricular septa (with a thickness greater than 15 mm). They also had dyspnea and shortness of breath. According to the proband, her grandmother died of sudden cardiac arrest and father (I-01, 38-years-old) died of traffic accident. Her young brother (II-03, 22-years-old) had no clinical symptoms.

The ventricular wall thickness or interventricular septum thickness of 30 patients with HCM were all greater than 15 mm. Most of these 30 patients had dyspnea and shortness of breath. Some with severe cases had syncope. At the same time, the 200 healthy controls showed normal hearts according to echocardiography and had no clinical symptoms.

### Variation identification in the HCM pedigree

Whole-exome sequencing was performed on the four people as shown in Fig. 1a. Approximately 50 GB data was obtained. The Q30 (the probability of an incorrect base call 1 in 1000 times) values of these four samples were all larger than 92%. After performing an analysis of variant segregation based on a family genetic model, we found a variant located in *MYH7* (NM_000257.4) in the patients but not in the healthy people of this pedigree. This variant was NM_000257.4:c.3134G > A (NP_000248.2:p.Arg1045His, rs397516178, c.3134G > A in short). To confirm the results of exome sequencing, the variants found in the exome were sequenced by Sanger sequencing in the four family members (Fig. 1c). All three patients (I-02, II-01 and II-02) carried the pathogenic mutation (heterozygotes). The other one healthy family members did not have the mutation.

### Frequency investigation of c.3134G > A by TaqMan custom SNP genotyping assays

We investigated the frequency of c.3134G > A in 200 healthy controls and 30 HCM patients. The variant was not found in the 200 healthy people and was found in only 1 of the 30 HCM patients. This patient had dyspnea and shortness of breath, and his ventricular wall was thickened (17 mm). According to his account, his mother had shortness of breath before she died.

### ACMG evaluation of variant sites

According to the American College of Medical Genetics and Genomics (ACMG) guidelines [12], c.3134G > A was evaluated. The variant was absent in our controls. Its frequency is 0.000048 (6/125568) in the TOPMED database and 0.00003 (1/31380) in the GnomAD database, and it is not found in Asian people based on the information in these databases. It meets the PM2 criterion of the ACMG guidelines. The functional effect of this variant was predicted by SIFT and PROVEAN [18]. The SIFT score was 0.000 (Damaging), and the PROVEAN score was -3.96 (Deleterious). Multiple computational predictions support a deleterious effect of this variant (PP3 criterion of the ACMG guidelines). The p.Arg1054Leu variant is classified as pathogenic in the ClinVar database (ClinVar Accession: RCV000629019.2; PM5 criterion of the ACMG guidelines). This variant cosegregates with the disease phenotype and the two affected family members in our pedigree (PP1 criterion of the ACMG guidelines).

## Discussion

Since Tanigawa and Jarcho et al. completed sequencing analysis of a family of patients with hypertrophic cardiomyopathy at the end of the last century [19], successive studies have confirmed that missense mutations of the *MYH7* gene can lead to the development of hypertrophic cardiomyopathy [20]. Since then, many variants have been found in the *MYH7* gene. Many pathogenic variants were found in this gene according to the ClinVar database, and many conflicting interpretations emerged. Therefore, it is important to find more evidence for these variants. Determination of the relationships between variants and diseases is very important for molecular genetic diagnosis, prognosis, and risk assessment for patients. In this study, we examined a family in which the carrier of c.3134G > A cosegregated with HCM. This variant was reported in several Italian HCM patients [21, 22]. In another study [23], c.3134G > A occurred in an American patient with early onset dilated cardiomyopathy (DCM). This patient also carried the Tyr5His variant in *TNNC1*. Because the author could not assess the segregation in the family, it is difficult to clarify the relationship between the mutations and disease. Therefore, determining the pathogenicity of variants is valuable for genetic counseling, even in relation to different heart diseases.

Finding c.3134G > A in more HCM families, especially families with different genetic backgrounds, and verifying cosegregation with the disease are very important to determine the pathogenicity of this variant. According to the PP1 criterion of the ACMG guidelines, more cases of cosegregation were found in people with diverse ethnic backgrounds. The criterion can be taken as moderate or strong evidence. Therefore, PP1 may be taken as moderate evidence for PM because the c.3134G > A variant was found in different pedigrees with different ethnic genetic backgrounds. Finally, the evidence for PM2, PP3, PM5 and PM (upgraded from PP1) supports c.3134G > A as pathogenic. Based on the ACMG guidelines, the c.3134G > A variant should be classified as "Likely pathogenic".

*MYH7* encodes beta myosin heavy chain (MHC-β), which is the major protein comprising the thick filament in cardiac muscle. Muscle myosin contains 2 heavy chain subunits, 2 alkali light chain subunits, and 2 regulatory light chain subunits. Mutations in the *MYH7* gene may reduce the ability of myosin to slide along actin filaments and impair the function of heart muscle. The variant p.Arg1045His is located in the S2-region of myosin. The S2 region of myosin contains many disease-associated variants. It is the binding site for MyBP-C, suggesting that binding between myosin S2 and MyBP-C is important for the development of HCM [24]. Further research on how these mutations influence the binding of myosin S2 and MyBP-C would still be very valuable.

## Conclusions

These studies not only further clarify what kind of role the S2 region plays in muscle contraction but also provide an important basis for the demonstrated relationship between c.3134G > A and HCM.Our findings suggest that this variant may be closely related to the occurrence of the disease. According the ACMG guidelines, the c.3134G > A variant should be classified as "Likely pathogenic".

## Data Availability

The raw DNA datasets used and analysed during the current study are deposited in NCBI database (Accession ID: PRJNA735333), https://www.ncbi.nlm.nih.gov/sra/PRJNA735333.

## Abbreviations

HCM: Inherited hypertrophic cardiomyopathy
ICM: Inherited cardiomyopathy
*MYH7*: Cardiac β-myosin heavy chain
ACMG: American College of Medical Genetics
SMC: Structural maintenance of chromosomes
